# GMTI for Squint Looking XTI-SAR with Rotatable Forward-Looking Array

**DOI:** 10.3390/s16060873

**Published:** 2016-06-14

**Authors:** Kai Jing, Jia Xu, Zuzhen Huang, Di Yao, Teng Long

**Affiliations:** School of Information and Electronics, Beijing Institute of Technology, Beijing 100081, China; jakey@bit.edu.cn (K.J.); hzzhit@126.com (Z.H.); ddyao@bit.edu.cn (D.Y.); longteng@bit.edu.cn (T.L.)

**Keywords:** synthetic aperture radar (SAR), ground moving target indication (GMTI), squint looking, cross-track interferometry (XTI), along-track interferometry (ATI), parameter estimation, displaced phase center array (DPCA)

## Abstract

To realize ground moving target indication (GMTI) for a forward-looking array, we propose a novel synthetic aperture radar (SAR) system, called rotatable cross-track interferometry SAR (Ro-XTI-SAR), for squint-looking application in this paper. By changing the angle of the cross-track baseline, the interferometry phase component of squint-looking Ro-XTI-SAR caused by the terrain height can be approximately adjusted to zero, and then the interferometry phase of Ro-XTI-SAR is only sensitive to targets’ motion and can be equivalent to the along track interferometry SAR (ATI-SAR). Furthermore, the conventional displaced phase center array (DPCA) method and constant false alarm (CFAR) processing can be used to accomplish the successive clutter suppression, moving targets detection and relocation. Furthermore, the clutter suppressing performance is discussed with respect to different system parameters. Finally, some results of numerical experiments are provided to demonstrate the effectiveness of the proposed system.

## 1. Introduction

It is well known that the along-track interferometry synthetic aperture radar (ATI-SAR) has been well studied in the past several decades for surface motion sensing [1,2,3,4,5,6,7] and ground moving target indication (GMTI) [8,9,10,11,12,13,14,15]. The interferometry phases among multiple along-track receivers are sensitive to the target’s radial motion, and they are used to cancel the static background clutters. Meanwhile, the cross-track interferometry synthetic aperture radar (XTI-SAR) is usually used for the digital elevation model (DEM) generation [16,17,18] in the side-looking application because the interferometry phases among cross-track multiple receivers are sensitive to the target’s height. Nevertheless, few studies can be found on GMTI for XTI-SAR, though many real systems with cross-baselines still only have strong demands on the GMTI [19,20,21,22,23,24,25,26,27,28]. For example, the airborne navigation or fire-control radars are normally mounted on the plane nose with a forward-looking array antenna, where the receivers are all distributed in the plane perpendicular to the flying track. Obviously, it is attractive but challenging to realize GMTI function for XTI-SAR.

Normally, different from the conventional side-looking applications, squint-looking or forward-looking applications is usually preferred for a radar equipped with a forward-looking array antenna. It may also provide the feasibility as well as an effective solution to accomplish GMTI via XTI-SAR. It is found that the interferometry phase among receivers of squint looking XTI-SAR may be jointly sensitive to terrain height and target motion. In addition, the sensitivity of terrain height is mainly decided by the cross-track baseline angle to the ground plane. Therefore, we propose a novel rotatable XTI-SAR (Ro-XTI-SAR) system to adjust the baseline angle by rotating the antenna array. After this step, the interferometry phase may be only sensitive to the motion of target, approximately. Thus, the conventional displaced phase center array (DPCA) and constant false alarm (CFAR) method can be used to suppress the strong ground clutters and automatically extract moving targets, respectively. Furthermore, the shifted location of target can be determined by estimating target radial velocity by using Ro-XTI-SAR with three receivers. The complete design and flowchart of Ro-XTI-SAR is provided in this paper for moving target detection, motion estimation and relocation. Furthermore, by introducing the clutter suppression factor, GMTI performance is discussed with respect to the system parameters as baseline, squint angle, swath and platform altitude. Finally, the results of numerical experiments are provided to demonstrate the effectiveness of the proposed method. Therefore, the main contribution of this paper is to propose an effective clutter suppressing and GMTI method for the squint-looking XTI-SAR, which is normally regarded as challenging and seldom investigated in the existing literatures.

The remainder of this paper is arranged as follows. In Section 2, the interferometry phases of ATI-SAR and XTI-SAR are analyzed as well as squint-looking Ro-XTI-SAR for a moving target with height and velocity. In Section 3, the system performance is discussed for terrain clutter suppression with respect to different factors. In Section 4, some results of numerical experiments are provided to demonstrate the effectiveness of the proposed method. In Section 5, some conclusions are drawn.

## 2. Rotatable Cross-Track Interferometry SAR (Ro-XTI-SAR)

### 2.1. Along-Track Interferometry SAR (ATI-SAR)

**Figure 1:**
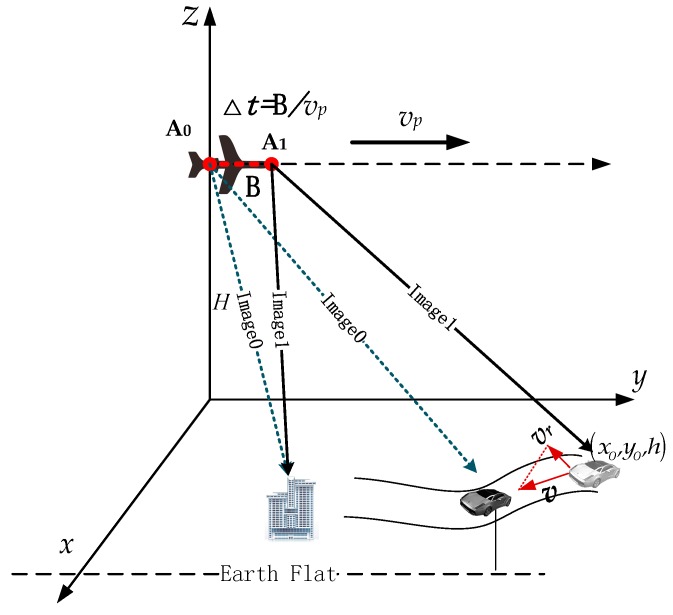
Side looking airborne ATI-SAR system geometry model.

For an along track interferometry SAR (AIT-SAR) system such as Figure 1, let us define a coordinate system ∠xyz, where the *x*-axis is the cross track direction, and the *y*-axis is the the along track (azimuth) direction. The multiple channel antennas of the radar are assembled at the side of the aircraft. The images of fixed targets acquired by two channels A_0_ and A_1_ are co-registered to the same position with the same responses, while the moving target will also focus with an approximately same position between images. Obviously, to effectively measure the slow moving targets’ motion in ATI-SAR images, three main procedures should be done, *i.e.*, the static clutter suppression, the moving target detection and the parameter estimation. Define a general point target (x,y,h) with the velocity v, radial velocity $v_{r}$ and height *h* as shown in Figure 1. *Image0* and *Image1* in Figure 1 are the focused images of channels A_0_ and A_1_, respectively. There is a time delay $\Delta t=B/v_{p}$ between two images, which causes the differences in complex responses between the moving and static targets. The response in each channel for a static/moving target in complex image domain could be unified if written as

(1) $$\begin{array}{l} S_{0}(x,y,h,v_{r})=a_{s}\exp\left\{j\varphi_{0}\left(x,y,h,v_{r}\right)\right\}\\ S_{1}(x,y,h,v_{r})=a_{s}\exp\left\{j\varphi_{0}\left(x,y,h,v_{r}\right)+j\Delta \varphi \left(x,y,B\right)+j\phi \left(x,y,h,v_{r},B\right)\right\} \end{array}$$

where $a_{s}$ is the consistent amplitude response of the target in each channel, and B is the length of baseline vector $\vec{A_{0}A_{1}}$ in Figure 1. $\varphi_{0}$ is the phase response for the reference channel A_0_, and Δφ is the phase difference caused by location difference and response unbalancing between two channels, which can be compensated by the image co-registration and channel balancing processing [12,24]. ϕ is the interferometry phase caused by the displaced receive antenna, which is a function of the baseline, the target position and the motion parameters.

For the side-looking ATI-SAR, the interferometry phase has no sensitivity to the target heights with the expression from [6,10] as

(2) $$\phi =\frac{2\pi }{\lambda }\frac{v_{r}}{v_{p}}B$$

where B is the length of the along-track baseline, $v_{p}$ is the velocity of platform and λ is the wavelength. It obvious that the interferometry phase ϕ satisfy the follow equations as:

(3) $$\frac{\partial \phi }{\partial h}=0\text{ and }\phi |{}_{\left(v_{r}=0\right)}=0$$

Accordingly, the Equation (1) could be rewritten as

(4) $$\begin{array}{l} {\tilde{S}}_{0}(x_{0},y_{0},h,v_{r})=a_{s}\exp\left\{j\varphi_{0}\right\}\\ {\tilde{S}}_{1}(x_{0},y_{0},h,v_{r})=a_{s}\exp\left\{j\varphi_{0}+j\phi_{\text{ATI}}\left(x_{0},y_{0},v_{r},B\right)\right\} \end{array}$$

where $\phi_{\text{ATI}}$ is the interferometry phase and φ satisfies the condition in Equation (3) regardless of terrain height.

### 2.2. Cross Track Interferometry Phase in Squint Mode

**Figure 2:**
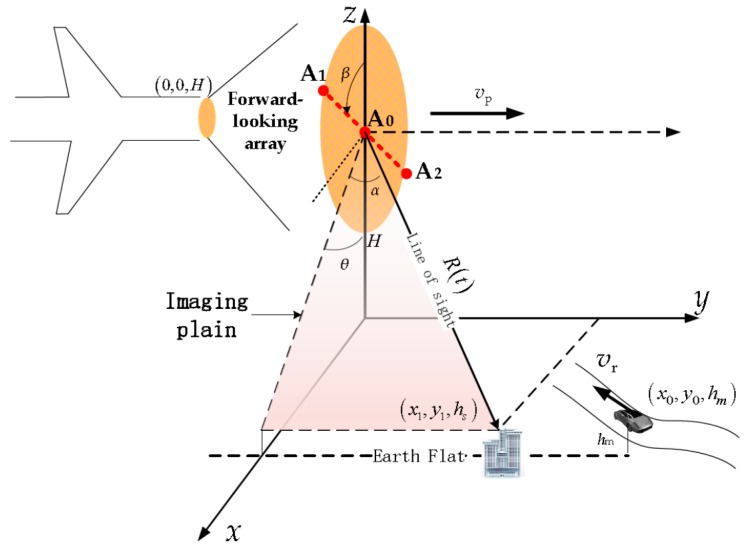
Airborne squint looking XTI-SAR system.

An airborne squint-looking three-channel XTI-SAR system is shown in Figure 2. The *x*-axis is the cross track (range) direction, and the *y*-axis is the along track (azimuth) direction, and α is the squint-looking angle of the XTI-SAR. The forward-looking array antennas of the radar are assembled in the front of the aircraft. The platform flies with the constant velocity $v_{p}$ torwards the *y*-axis in the height of *H*. A moving target locates at $\left(x_{0},y_{0},h_{m}\right)$ while a static target locates at $\left(x_{1},y_{1},h_{s}\right)$ when t=0. The moving target keeps a constant radial velocity $v_{r}$.

The signal model of XTI-SAR has been widely studied like Rosen *et al.* [15], Madsen [16], Zebker and Goldstein [16,18], Li [17] and Graham [22]. Graham [22] analyzed the XTI-SAR and obtained the phase response for squint-looking and side-looking XTI-SAR. Here, we started from the interferometry phase Expression (30) in [15] of the XTI-SAR system, from which the interferometry phase between two channels for a static target with height of *h_s_* can be given as:

(5) $$\begin{array}{cc} \phi_{\text{XTI}}(R,\theta ,\beta ,h_{s},B) & =\frac{2\pi }{\lambda }B\frac{\cos(\theta -\frac{\pi }{2}+\beta )}{R\sin(\theta )}h_{s}\\ & =\frac{2\pi }{\lambda }B\frac{\sin(\theta +\beta )}{R\sin(\theta )}h_{s} \end{array}$$

where B is the length of baseline $\vec{A_{0}A_{1}}$ in Figure 2, R is the slant range when *t* = 0, *θ* is the incidence angle, and β is the baseline angle of the cross-track baseline in the plain which is perpendicular to *y*-axis or platform velocity vector. The validity of Equation (5) has been demonstrated in [15], which shows Equation (5) is not only suitable for side-looking but also for squint looking mode.

However, the phase of Equation (5) is derived based on the static target without taking the target’s motion into consideration. Furthermore, the interferometry phase of the cross track baseline caused by the target’s motion in squint looking is also different from the static clutters from [14,16,21,23]. In this case, with consideration of squint looking, the interferometry phase Expression (5) should be rewritten as

(6) $$\phi_{\text{sXTI}}(R,\theta ,\beta ,\alpha ,h_{m},v_{r},B)=\varphi_{\text{XTI}}(R,\theta ,\beta ,h_{m},B)+\psi (\theta ,\beta ,\alpha ,v_{r},B)$$

where ψ is the interferometry phase caused by the target’s velocity. The phase ψ is also related to the baseline *B*, the squint angle α, the incidence angle θ and the baseline angle β. According to [15,23], the ψ can be further expressed as

(7) $$\begin{array}{cc} \psi (\theta ,\beta ,\alpha ,v_{r},B) & =-\frac{2\pi }{\lambda }\frac{v_{r}y_{1}}{v_{p}x_{1}}B\sin\beta \\ & =-\frac{2\pi }{\lambda }\frac{v_{r}\tan\alpha }{v_{p}\sin\theta }B\sin\beta \end{array}$$

### 2.3. The Proposed Ro-XTI-SAR System for Clutter Suppression

By combining Equations (5) and (7), the interferometry phase of a general target for the squint looking XTI-SAR system can be further arranged as:

(8) $$\begin{array}{l} \phi_{\text{sXTI}}(R,\theta ,\beta ,\alpha ,h,v_{r},B_{c})=\varphi_{\text{XTI}}+\psi \\ =\frac{2\pi }{\lambda }B\frac{\sin(\theta +\beta )}{R\sin(\theta )}h-\frac{2\pi }{\lambda }\frac{v_{r}\tan\alpha }{v_{p}\sin\theta }B\sin\beta \end{array}$$

By comparing Equations (8) and (2), it is easy to find that the $\phi_{\text{sXTI}}$ could be equivalent to $\phi_{\text{ATI}}$ by forcing the value of $\varphi_{\text{XTI}}$ to zero. This means the squint looking XTI-SAR system could be equivalent to an ATI-SAR system. From Equation (5), it can be done by adjusting the baseline angel β to meet

**Figure 3:**
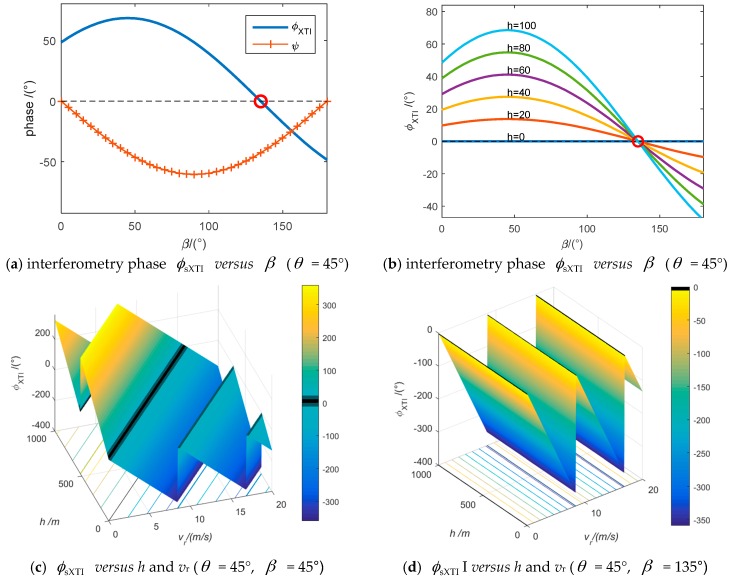
Squint-looking XTI-SAR interferometry phases of different baseline angles. (**a**) When *h* = 50 m, *v_r_* = 1 m/s and θ = 45° the interferometry phase of static target is forced zero when *β* = 135°, the interferometry phase with respect to target’s height is non-zero and could be used to estimate the velocity; (**b**) It is demonstrated that the interferometry phase $\phi_{\text{XTI}}$ caused by the static targets with different height *h* are all forced to zeros when β = 180 − θ; (**c**) When θ = 45° and β = 45°, *i.e.*, sin(θ+β)≠0, the interferometry phase $\phi_{\text{XTI}}$ is jointly decided by terrain height and target radial velocity; (**d**) When θ = 45° and β = 135°, *i.e.*, sin(θ+β)=0, the interferometry phase $\phi_{\text{XTI}}$ is constant regardless of height *h* and it is linearly varied with target’s radial velocity *v*_r_. That is, the interferometry phase will be linearly related to the target’s radial velocity *v*_r_.

(9) sin(θ+β)=0

which means θ+β=kπ,(k∈N) or the baseline is parallel to the imaging plain of the target. We designed some experiments to demonstrate the effectiveness of Equation (9). The results have been shown in Figure 3. The simulation parameters are designed according to an X-band airborne squint looking XTI-SAR system. The altitude H of the platform is 5000 m, the velocity $v_{p}$ is 139 m/s, the baseline length B is 0.45 m and the squint angle α = 45°. 

Therefore, when sin(θ+β)=0, the terrain clutters of the scene for the XTI-SAR could be cancelled by the DPCA method used in the ATI-SAR processing, and the relationship between the interferometry phase and radial velocity could be derived as:

(10) $$\begin{array}{cc} \phi_{\text{sXTI}} & =\psi \left(v_{r}\right)=-\frac{2\pi }{\lambda }\frac{v_{r}\tan\alpha }{v_{p}\sin\theta }B\sin\left(\pi -\theta \right)\\ & =-\frac{2\pi }{\lambda }\frac{v_{r}}{v_{p}}B\tan\alpha \end{array}$$

**Figure 4:**
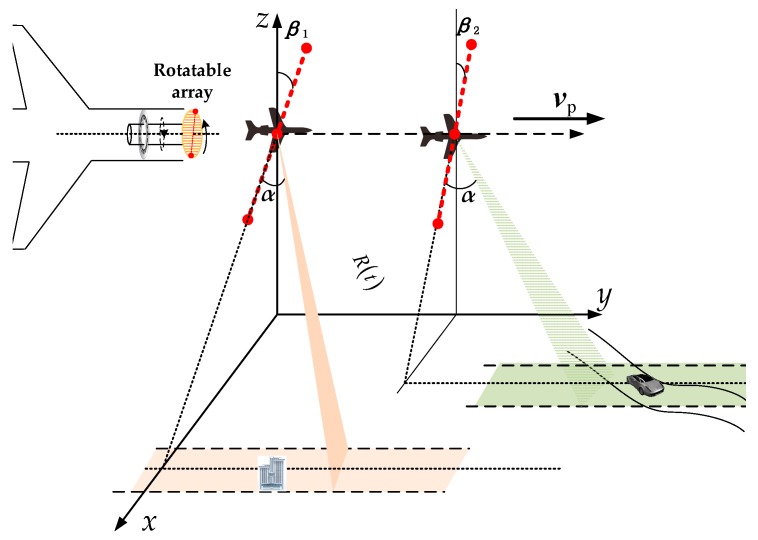
Ro-XTI-SAR system model with the rotatable forward-looking array.

It is obvious that the interferometry phase $\phi_{\text{sXTI}}$ is a monotonically increasing function with the squint angle α, which means that the proposed system works better with increased squint angle in the view of interferometry processing. To meet θ+β=kπ,(k∈N), the rotatable XTI-SAR (Ro-XTI-SAR) system is proposed in this paper. The multiple channel antennas of the Ro-XTI-SAR are assembled in the front of the aircraft with a rotatable turn plate as shown in Figure 4. Then, the baseline angle β between the baseline and the altitude direction could be controlled to adjust the interferometry sensitivity to the terrain height—specifically, adjusting the baseline angle β to meet θ+β=kπ,(k∈N) for a synthetic aperture time. GMTI can be accomplished subsequently with the ATI-SAR method. Furthermore, the moving target velocity estimation and relocation should be done after the clutter cancellation and target detection. When the center of imaging area changes, the Ro-XTI-SAR adjusts the baseline angle β again, and it will be fixed during the synthetic aperture time for imaging.

### 2.4. The Complete Flowchart of the Ro-XTI-SAR System

**Figure 5:**
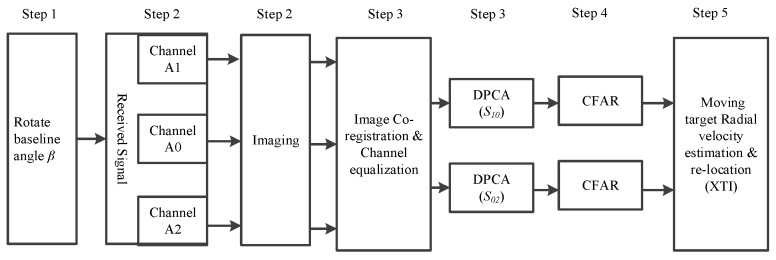
The signal processing flowchart of a three-channel Ro-XTI-SAR system.

A complete flowchart can now be given in Figure 5 for the proposed three-channel airborne Ro-XTI-SAR system as in Figure 4. The flowchart is introduced step-by-step as follows:

**Step 1:**  Determine the area of interest and then rotate the forward-looking array to make the baseline angle β satisfy the condition θ+β=kπ,(k∈N).
**Step 2:**  Collect the reflection signal from the scenario and obtain the focused images of each channel by well used SAR scene reconstruction algorithms.
**Step 3:**  Image co-registration and channel balancing are done as the pre-processing before DPCA for images of different channels. Then, the outputs of A0 and A1 channels are used to generate the DPCA result $S_{10}$, while the outputs of A0 and A2 channels are used to generate the DPCA result $S_{02}$ [26].
**Step 4:**  Moving target detection is accomplished by CFAR detector on the DPCA image.
**Step 5:**  Estimate the motion and position parameter of each detected targets as follows:

The interferometry phase of the moving targets could be obtained as:

(11) $${\tilde{\phi }}_{\text{sXTI}}=\mathrm{angle}\left(S_{10}\cdot \bar{S_{02}}\right)$$

Then, the equation of estimated target velocity could be derived from Equation (10) as:

(12) $${\tilde{v}}_{r}=-\frac{\lambda v_{p}}{2\pi B\tan\alpha }{\tilde{\phi }}_{\text{sXTI}}$$

and the azimuth shift (*y*-axis) [6,9] is

(13) $$\Delta y=-\frac{{\tilde{v}}_{r}}{v}R$$

From [23,24], the moving targets will shift towards the direction across the radar line of sight (RLOS). Therefore, the cross track shift due to the presence of squint could be determined as:

(14) $$\Delta x=-\Delta y\frac{{\hat{y}}_{0}}{{\hat{x}}_{0}}$$

where ${\hat{x}}_{0}$ and ${\hat{y}}_{0}$ are the focus position of the moving target.

## 3. Performance Limitations and Design Rules of Ro-XTI-SAR

### 3.1. Performance Limatitions Due to the Incidence Angle Difference

However, it should be noticed that only the terrain clutters near the beam center could be suppressed completely and the targets located away from the beam center will have a baseline angle difference. As a result, the clutter suppressing performance will also be reduced. In order to evaluate this kind of performance loss, we define the clutter suppression factor $\eta_{c}$ as the ratio of amplitude values of the target’s response in an image domain before and after and DPCA as:

(15) $$\eta_{c}=\frac{\left|{\tilde{S}}_{1}-{\tilde{S}}_{0}\right|}{\left|{\tilde{S}}_{0}\right|}$$

where ${\tilde{S}}_{0}$ and ${\tilde{S}}_{1}$ are the complex responses of a pixel in the image domain of A0 and A1 channels of Figure 2. By substituting Equations (4) and (5) into Equation (15), the suppression factor expression becomes

(16) $$\begin{array}{ll} \eta_{c} & =\left|\frac{a_{s}\exp\left\{j\varphi_{0}+j\phi_{\text{XTI}}\right\}-a_{s}\exp\left\{j\varphi_{0}\right\}}{a_{s}\exp\left\{j\varphi_{0}\right\}}\right|\\ & =\left|\exp\left\{j\phi_{\text{XTI}}\right\}-1\right|\\ & =\sqrt{{\left(1-\cos\phi_{\text{XTI}}\right)}^{2}+\sin^{2}\phi_{\text{XTI}}}\\ & =\sqrt{2\left(1-\cos\phi_{\text{XTI}}\right)}\\ & =2\left|\sin\frac{\phi_{\text{XTI}}}{2}\right| \end{array}$$

For the static target with the position $\left(R,\theta_{t},\alpha +\Delta \alpha \right)$, the incidence angle $\theta_{t}$ of the target is

(17) $$\theta_{t}=\theta +\Delta \theta$$

where θ is the beam pointing incidence angle, and Δθ is the difference of incidence angle. Then, the interferometry phase of the Ro-XTI-SAR would be derived from Equation (5) as:

(18) $$\begin{array}{cc} \phi_{\text{sXTI}} & =\phi_{\text{XTI}}=\frac{2\pi }{\lambda }B_{c}\frac{\sin(\theta +\Delta \theta +\beta )}{R\sin(\theta_{t})}h\\ & =\frac{2\pi }{\lambda }B_{c}\frac{\sin(\Delta \theta +\pi )}{R\sin(\theta_{t})}h=-\frac{2\pi }{\lambda }B_{c}\frac{\sin(\Delta \theta )}{R\sin(\theta_{t})}h \end{array}$$

Then, from Equations (16) and (18), the suppression factor of the static target with an incidence angle difference Δθ can be expressed as

(19) $$\eta_{c}=2\left|\sin\left(\frac{\pi }{\lambda }B_{c}\frac{\sin(\Delta \theta )}{R\sin(\theta_{t})}h\right)\right|$$

which is a function of the target incidence angle $\theta_{t}$, the wave length λ, the baseline length $B_{c}$, the incidence angle difference Δθ, the target height *h*, and the target range R. In addition, R could be represented as:

(20) $$R=\frac{\left(H-h\right)}{\cos\theta_{t}\cos\alpha }$$

### 3.2. The Proposed Ro-XTI-SAR Design Rules

**Figure 6:**
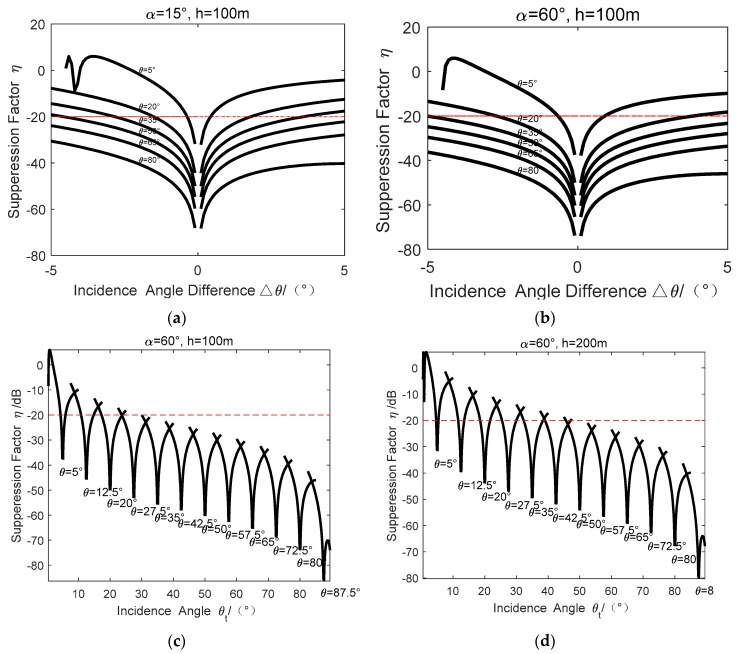
The suppression factor *versus* different system parameters. (**a**) is the suppression factor versus incidence angle difference when the target height is 100m and squint angle is 15°; (**b**) is the suppression factor versus incidence angle difference when the target height is 100m and squint angle is 60°; (**c**,**d**) are the suppression factor versus the incidence angle at different targets’ height. It’s found that the suppression factor becomes better with the increases of the incidence angle accordingly.

In this sub-section, we will demonstrate the effectiveness of derived suppression factor as Equation (19), which is helpful for optimizing the Ro-XTI-SAR system parameters as well as the ultimate GMTI performance. At first, let us take look at a simulated results based on Equation (19) of a squint looking airborne radar system with the system parameters of λ = 0.273 m, B = 0.45 m, H = 8 km and $\theta_{\text{beam}}$ = 10°. The squint angle α and the target height *h* are displayed in the figures. Figure 6a,b are the suppression factors *versus* the target incidence angle $\theta_{t}$ for different target heights, respectively. Figure 6c,d are the factors *versus* beam incidence angle θ and the incidence angle difference for different squint angles, respectively. Since the incidence angle differences between target and beam center should be less than half of the beam width, only the factor performance when $\Delta \theta =\theta -\theta_{t}\leq \theta_{\text{beam}}/2$ are plotted is these figures, where the beam pointing angle varied from 0°~90° with the interval 7.5°. Some conclusions could be drawn as:

(1) From Figure 6a,b, it is shown that when the incidence angle difference Δθ = 0, we have the suppression factor $\eta_{c}=0$. That is, the ground clutter can be cancelled successfully. However, for the other targets that have non-zero incidence angle differences, the suppression ability will be reduced. In addition, the clutter suppression ability will be improved with the increases of squint angle by comparing Figure 6a,b.
(2) From Figure 6c,d, it is found that the suppression factor becomes better with the increases of the incidence angle θ accordingly.
(3) With the parameters of Figure 6c with the target height of 100 m, when the incidence angle of the target is larger than 35°, the clutter suppression factors are all below −20 dB. Thus, this will usually be enough for the static clutter suppression. When the maximal terrain height increases to 200 m, the minimal incidence angle becomes 50°, as shown in Figure 6d. That is, clutter suppression ability will become worse with the increase of the target height.

From the above analysis, we can design system parameters by forcing the suppression factor $\eta_{c}$ below a desired limitation value $\eta_{\text{limit}}$, e.g., −20 dB as:

(21) $$\eta_{c}\leq \eta_{\text{limit}}\Rightarrow \left|\sin\left(\frac{\pi }{\lambda }B\frac{\sin(\Delta \theta )}{R\sin(\theta_{t})}h_{\max}\right)\right|\leq \eta_{\text{limit}}$$

Then, the design rule on the baseline of Ro-XTI-SAR could be derived as:

(22) $$\text{Rule}1:\;B\leq \frac{\lambda \text{arcsin}\eta_{\text{limit}}}{\pi h_{\max}\sin(\frac{\theta_{\text{beam}}}{2})}\frac{\left(H-h_{\max}\right)}{\cos\alpha }\tan(\theta_{t})$$

where $h_{\max}$ is the maximal height of the terrain, and $\theta_{\text{beam}}$ is the beam pitching width. Equation (22) means that the cross track baseline should not be too long for clutter cancellation. Otherwise, it also should not be too small to sustain the estimation accuracy of the velocity. Then, the design rule or the limitation on target incidence angle should meet

(23) $$\text{Rule}2:\;\theta_{t}\geq \text{arctan}\left(\frac{B_{c}\cos\alpha }{\lambda \text{arcsin}\eta_{\text{limit}}}\frac{\pi h_{\max}\sin(\frac{\theta_{\text{beam}}}{2})}{\left(H-h_{\max}\right)}\right)$$

It is shown that the Ro-XTI-SAR has better clutter cancellation performance for the area with a large incidence angle. Subsequently, the design rule or the limitation on terrain height is given as:

(24) $$\text{Rule}3\;:\;h_{\max}\leq \frac{H}{\left(1+B_{c}\frac{\pi \sin(\frac{\theta_{\text{swath}}}{2})}{\lambda \text{arcsin}\eta_{\text{limit}}}\frac{\cos\alpha }{\tan(\theta_{t})}\right)}$$

Equation (24) tells us that Ro-XTI-SAR has a limitation on the maximal terrain height. When the terrain height is higher than $h_{\max}$, the clutter suppressing ability cannot be guaranteed. Furthermore, the maximum incidence angle area of swath $\theta_{\text{swath}}$ should meet

(25) $$\text{Rule}4:\;\theta_{\text{swath}}\leq 2\text{arcsin}\left(\frac{\lambda \text{arcsin}\eta_{\text{limit}}}{\pi h_{\max}B_{c}}\frac{\left(H-h_{\max}\right)}{\cos\alpha }\tan(\theta_{t})\right)$$

In summary, with the derived suppressing ratio as Equation (19), the Ro-XTI-SAR systems could be optimally designed for obtaining better GMTI performance according to the rules above as Equations (22)–(25).

## 4. Numerical Experiments of the Proposed Ro-XTI-SAR

**Figure 7:**
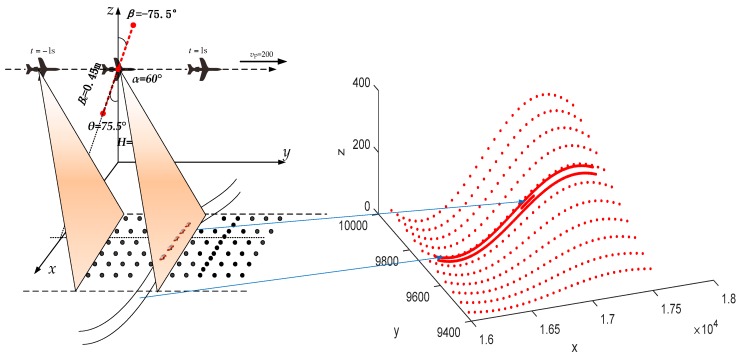
Ro-XTI-SAR system geometry and simulated targets.

In this section, we will investigate the performance of the proposed Ro-XTI-SAR system based on a simulated airborne system shown in Figure 7. Radar echoes of the scene are acquired by an airborne radar system working in the strip-mode with the carrier frequency 11 GHz, bandwidth 100 MHz, sampling frequency 150 MHz, platform velocity 200 m/s, pulse repetition frequency 1000 Hz. The length of the baseline is *B* = 0.45 m with three channels. The baseline angle *β* has been rotated to an angle of −75.5° towards the scene center. The squint angle α is 60°. The strip swath width is 1.5 km. Six cars move on a road with a slowly velocity as shown in Table 1. The heights of the terrain are all bellow 400 m from ground plane. The signal clutter ratio (SCR) of the targets are all −20 dB in the image domain.

**Figure 8:**
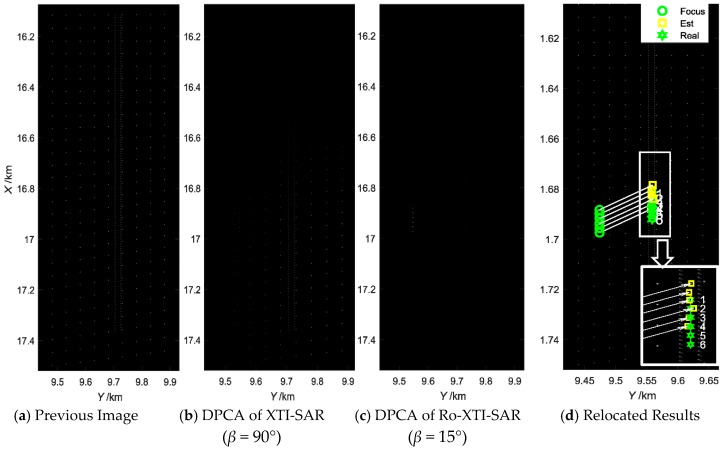
GMTI results of three-channel airborne Ro-XTI-SAR system. (**a**) is the previous SAR image; (**b**) is the image after DPCA of a traditional XTI-SAR with the baseline angle 90°; (**c**) is the image after DPCA of the proposed Ro-XTI-SAR system; (**d**) is the detection and relocation results of the moving targets.

The GMTI algorithm [25] is used to process the echoes. The previous SAR image without suppressing the stationary clutters is displayed in Figure 8a, and it is very difficult to detect the moving targets in the image because of the interference of the strong background clutter and the −20 dB low SCR. That is, the SCR needs to be increased to a large extent in order to detect the moving targets. First, by combining the Ro-XTI-SAR system (baseline angle *β* = −75°) and the DPCA method in image domain, the terrain stationary clutters could be suppressed as shown in Figure 8c. As a comparison, the DPCA results from a traditional XTI-SAR system with baseline angle *β* = 0° is displayed in Figure 8b, from which the clutters with height could not be canceled efficiently.

After terrain clutter suppression, the moving targets become outstanding in Figure 8c with about 45 dB SCR improvement factor. Furthermore, the estimation results via Figure 5 are displayed in Figure 8d, which indicates that the moving targets are relocated near their real positions in the scene. The circles indicate the detected target positions, and the rectangles indicate the relocated positions. Table 1 shows the estimation results of the moving targets, and the estimated radial velocities of the targets are precise, with little location errors caused by the targets’ height.

## 5. Conclusions

In this paper, a novel Ro-XTI-SAR system is proposed to realize the SAR/GMTI for the squint-looking applications only via forward-looking array. It is found that the XTI-SAR system could be converted into the conventional ATI-SAR by rotating the cross track baseline angle, and the interferometry phases will be sensitive to targets’ radial velocities regardless of scene height. Furthermore, the performance of the Ro-XTI-SAR system is discussed based on the clutter suppression factor. Although the clutter suppressing performance will be reduced for the targets away from the beam center, the GMTI performance on the edge of the imaging swath can be acceptable by optimizing system geometry design. In addition, some rules are proposed for system parameter design of the proposed Ro-XTI-SAR system. Finally, some simulated results are provided to demonstrate the effectiveness of the proposed method.

## Figures and Tables

**Table 1:** The estimation results of each targets.

Para.	Target 1	Target 2	Target 3	Target 4	Target 5	Target 6	Mean Squ. Err.
x (m)	16,821	16,841	16,860	16,881	16,901	16,920	**38.4**
$\hat{x}$ (m)	16,785	16,804	16,822	16,840	16,861	16,880	
y (m)	9717	9717	9717	9717	9717	9717	2.18
$\hat{y}$ (m)	9718	9716	9716	9721	9715	9714	
h (m)	128	135	142	149	155	161	**145.5**
$\hat{h}$ (m)	--	--	--	--	--	--	
$v_{r}$ (m/s)	1.7317	1.7323	1.7328	1.7333	1.7338	1.7343	0.060
${\hat{v}}_{r}$ (m/s)	1.6952	1.6716	1.6730	1.7144	1.6619	1.6485	
